# Overall health knowledge in the Philippines, multilevel confirmatory factor analysis of the Philippine National Demography Health Survey 2022 data

**DOI:** 10.1038/s41598-024-68819-4

**Published:** 2024-08-12

**Authors:** Nesma Lotfy

**Affiliations:** Department of Biostatistics, High Institute of Public Health, Alexandria University, Alexandria, Egypt

**Keywords:** Health knowledge, ML-CFA, The Philippines, DHS, Health care, Medical research

## Abstract

A secondary data analysis of the 2022 Philippine National Demographic and Health Survey (PNDHS) was conducted to explore the underlying structure of knowledge regarding communicable and noncommunicable diseases using multilevel confirmatory factor analysis (CFA). The PNDHS data consist of two levels: level-1 represents within-household data (household questionnaire), and level-2 represents between-household data (primary sampling unit (PSU)). Therefore, a two-level CFA and two-level variance CFA were performed. Furthermore, a multigroup analysis assessed the structural differences between males/females and urban/rural groups. In the PNDHS survey, 30,372 household interviews were completed. Knowledge levels for cancer, heart disease, diabetes, dengue fever, TB, and COVID-19 were 96.7%, 94.9%, 97.8%, 98.4%, 96.7%, and 92.8%, respectively. The two-level CFA indicated that the coefficient loadings of each item for both levels were statistically significant (Z-test, P < 0.001). Regarding two-level variance CFA, the variance at level-1 was higher than that at level-2 (13 and 6.7, respectively). The multigroup analysis revealed that the model was non-invariant (not equal) across gender and residence (likelihood ratio test; P < 0.001, P < 0.001, respectively). In conclusion, level-1 has greater effect than does level-2 because the variance in level-1 is greater than that in level-2, the knowledge of COVID-19 has the lowest loading compared to other items, and rural/urban areas and females/males exhibit different levels of health knowledge.

## Introduction

Causes of death can be grouped into three categories: communicable, noncommunicable and injuries. The majority of deaths worldwide are caused by chronic illnesses (CDs), which are concentrated in low- and middle-income countries (77%)^1^. Globally, 7 of the 10 leading causes of death in 2019 were noncommunicable diseases. On the other hand, deaths from communicable diseases ranked 4th among the leading causes of death^2^.

In the Philippines, in 2020, ischaemic heart disease was the leading cause of death (18.5%), cerebrovascular disease was the second leading cause (10.3%), and cancer was the third leading cause (10.2%). Additionally, deaths due to diabetes mellitus account for approximately 6.4% of the total deaths, while deaths due to hypertensive diseases, which rank fifth, account for 5.7% of the total deaths in the country^3^. Moreover, according to the CDC, within the United States, 71% of reported TB cases are among non-U.S. born persons. Nearly 12.5% of these cases involved individuals born in the Philippines^4^. Finally, dengue fever is a communicable disease that is endemic in the Philippines, and the risk of transmission is highest during and immediately following the rainy season^5^.

Health knowledge is considered one of the most important issues in healthcare and public health^6^. A high level of health knowledge is associated with positive health behaviours, better health outcomes^7^, and lower healthcare costs^8^. Moreover, a good level of health knowledge contributes to improving global health in the general population^9^. In contrast, low health knowledge is associated with a range of adverse health effects, such as increased mortality^7^, increased rates of chronic diseases, the adoption of adverse health behaviours (smoking, alcohol consumption, and the use of illicit substances, sedentary lifestyles), and increased healthcare costs^10^. However, in the Philippines, the nationwide prevalence of poor health knowledge was 51.5%, while subnational prevalence estimates ranged from 48.2 to 65.4%^11^.

Knowledge about communicable and noncommunicable diseases was collected from the 2022 Philippine National Demographic and Health Survey (PNDHS)^12^ to formulate policies and improve public health programs. The PNDHS dataset is distinguished from single-level dataset by the nesting of observations within higher-level groups. Concerning statistical analysis, multilevel analysis (MLA) is an attractive approach for dealing with the PNDHS dataset where data are nested. MLA is used for studying the relationships between individuals and their various social groups because it allows the incorporation of substantive theory about individual and group processes into the sampling schemes of many research studies (e.g., multistage stratified samples, repeated measures designs) or into hierarchical data structures found in many existing datasets encountered in social science, management, and health-related research^13^.

Improving health knowledge plays an important role in increasing awareness of health care, which not only helps people know more about diseases and increases their ability for self-care but also helps them to avoid unhealthy behaviours and adopt healthy lifestyles^14^. Thus, the aim of the current study was to confirm the underlying structure of knowledge about these diseases using multilevel confirmatory factor analysis and to determine whether the structure differs according to gender or residence.

## Materials and methods

### Data source

A secondary data analysis was conducted on data from the household questionnaire of the 2022 PNDHS, which is the seventh Demographic and Health Survey (DHS) conducted in the Philippines in collaboration with The DHS Program. The PNDHS is a nationally representative survey that has been conducted every 5 years since 1968, and 2022 is the latest of the PNDHS conducted so far. The 2022 Philippine National Demographic and Health Survey (PNDHS) was implemented by the Philippine Statistics Authority (PSA). The survey was conducted from May 2 to June 22, 2022. The dataset can be obtained from the DHS program website upon authorization (https://dhsprogram.com/data/available-datasets.cfm). The sample selection methodology for the 2022 PNDHS was based on a two-stage stratified sample design. The first stage involved a systematic selection of 1247 primary sampling units (PSUs) distributed by province. In the second stage, an equal take of either 22 or 29 sample housing units were selected from each sampled PSU using systematic random sampling. The survey in 2022 resulted in 30,372 completed household interviews^12^.

### Measures

Health knowledge was measured through six items, and each item was rated as yes or no (“Do not know” answers were added to “No” answers for the last item). The items were

1. Have you ever heard of a disease called cancer?
2. Have you ever heard of heart disease?
3. Have you ever heard of diabetes?
4. Ever heard of dengue fever?
5. Have you ever heard of an illness called tuberculosis or TB?
6. Can COVID-19 infection be prevented?

### Ethics approval

The survey protocol was reviewed by the ICF Institutional Review Board (IRB number: 2022-021), and it was determined that the activities described were not human subjects research (NHSR).

### Data analysis

#### Descriptive statistics

Weighted percentages were estimated for each item in health knowledge.

#### Modelling

The PNDHS data are two-level data; level-1 is the household questionnaire (within-households or health knowledge), and level-2 is the primary sampling unit (PSU) (between-households). Three models were implemented:

##### Two-level confirmatory factor analysis

The confirmatory factor analysis was extended to account for the nested data as the households are nested through the PSU (group of households). The first latent variable was added to account for “health knowledge” (within-households), while the second latent variable was added at the “PSU” level (between-households) to account for possible PSU-PSU effects (constant within PSU and varying across PSU). The means of both latent variables were centered at zero^15^.

##### Two-level variance component confirmatory factor analysis

The previous model did not include any constraints, while in this model, it was assumed that the (unstandardized) factor loadings to be equal across both levels and freely estimated the factor variances at both levels^15^.

##### Multigroup analysis

Two series of multigroup multilevel CFA models were fit for gender (male/female) and residence (urban/rural). Implementation began by estimating a model where all parameters were freely estimated (unconstrained model), which was compared with a model that included equality-constrained loading and intercepts (constrained model). The likelihood ratio test was used to compare these two models to gauge the significance of the added restrictions^15^.

#### Model estimation

To model dichotomous data, the generalized structural equation model (GSEM), which is based on the integration of two generalized linear model (GLM) and structural equation modelling (SEM) algorithms, was used to confirm the underlying models using the Bernoulli distribution with logit link and the maximum likelihood method for estimating parameters. Unlike SEM, GSEM does not require the normality assumption^15,16^. All computations were performed using STATA version 16.0^17^.

## Results

**Figure 1:**
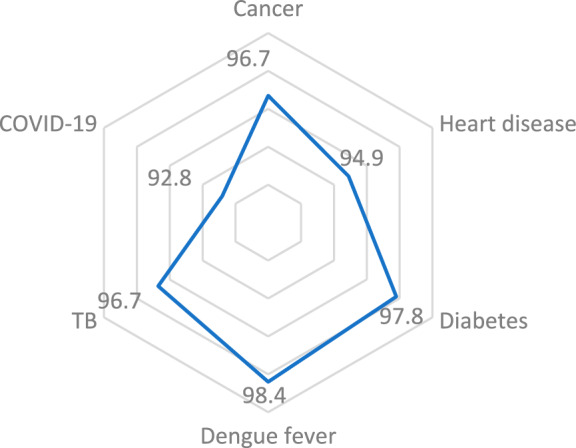
The weighted percentage of the health knowledge, the Philippines, 2022.

The percentage of individuals with knowledge of cancer, heart disease, diabetes, dengue fever, TB, and COVID-19 were 96.7%, 94.9%, 97.8%, 98.4%, 96.7%, and 92.8%, respectively (Fig. 1).

There is almost no difference in health knowledge between males and females. In terms of residence, health knowledge in rural areas was generally lower than that in urban areas. Specially, knowledge of heart disease and COVID-19 was 93.7% and 91.7%, respectively, in rural areas compared to 96.0% and 93.8%, respectively, in urban areas. (Table 1).

**Table 1:** The weighted percentage of health knowledge regarding gender and residence, the Philippines, 2022.

Variable	Cancer%	Heart disease%	Diabetes%	Dengue fever%	TB%	COVID-19%
Gender						
Male	96.6	94.8	97.9	98.2	96.4	93.1
Female	96.7	95	97.7	98.5	96.7	92.7
Residence						
Urban	97.0	96.0	98.2	98.8	97.3	93.8
Rural	96.2	93.7	97.3	98	95.9	91.7

Table 2 shows the unstandardized factor loadings for the two-level CFA and variance CFA. According to the two-level CFA, the coefficient loadings of each item on the within-household and between-household latent variables were all statistically significant (Z-test, P < 0.001). The within-household loadings are almost equal to the between-household loadings for all items except for COVID-19 (within-household = 0.14 and between-household = 0.26). Knowledge about diabetes had the maximum influence on the two latent variables, while knowledge about COVID-19 had the minimum effect.

**Table 2:** Unstandardized factor loading of the multilevel confirmatory factor analysis, the Philippines, 2022.

Items	Two-level CFA		Two-level variance CFA	
	Within-households	Between-households	Within-households	Between-households
Cancer	0.67*	0.72*	0.7*	0.7*
Heart disease	0.82*	0.8*	0.79*	0.79*
Diabetes	1.1*	1.1*	1.1*	1.1*
Dengue fever (constrained)	1	1	1	1
TB	0.61*	0.67*	0.64*	0.64*
COVID-19	0.14*	0.26*	0.19*	0.19*
Variance	14	6.3	13	6.7

*Z-test, P value < 0.001.

**Figure 2:**
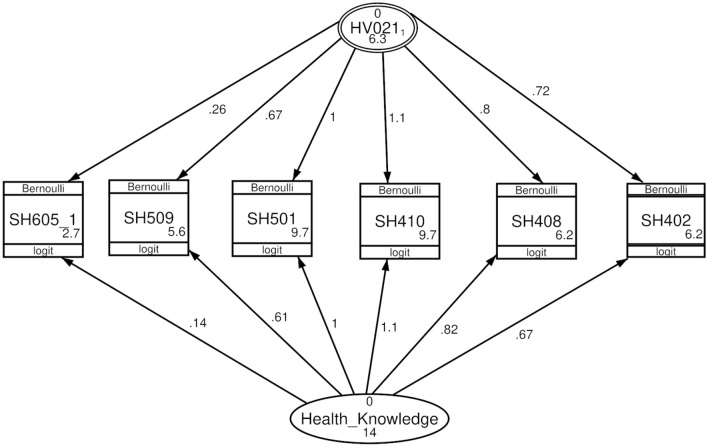
Two-level confirmatory factor analysis, the Philippines, 2022. SH402: Have you ever heard of a disease called cancer? SH408: Have you ever heard of heart disease? SH410: Have you ever heard of diabetes? SH501: Ever heard of dengue fever? SH509: Have you ever heard of an illness called tuberculosis or TB? SH605_1: Can COVID-19 infection be prevented? Health_Knowledge: level-1 (within-households). HV201: level-2 (between-households (PSU)).

**Figure 3:**
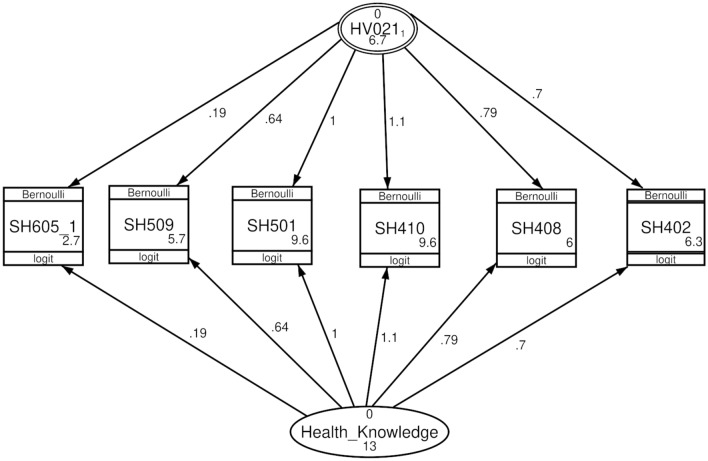
Two-level variance confirmatory factor analysis, the Philippines, 2022. SH402: Have you ever heard of a disease called cancer? SH408: Have you ever heard of heart disease? SH410: Have you ever heard of diabetes? SH501: Ever heard of dengue fever? SH509: Have you ever heard of an illness called tuberculosis or TB? SH605_1: Can COVID-19 infection be prevented? Health_Knowledge: level-1 (within-households). HV201: level-2 (between-households (PSU)).

According to the two-level variance CFA, the variance within-households was higher than the variance between-households (13 and 6.7, respectively). Figures 2 and 3 show the diagrams of the two-level CFA and two-level variance CFA.

Tables 3 and 4 show the testing of measurement invariance across gender and residence, respectively. According to the likelihood ratio test, the model is non-invariant (not equal) across gender and residence (likelihood ratio test; P < 0.001, P < 0.001, respectively).

**Table 3:** Multigroup multilevel confirmatory factor analysis for gender, the Philippines, 2022.

Items	Unconstrained model				Constrained model	
	Male		Female			
	Within-households	Between-households	Within-households	Between-households	Within-households	Between-households
Cancer	0.6*	0.7*	0.7*	0.73*	0.66*	0.71*
Heart disease	0.77*	0.78*	0.83*	0.81*	0.82*	0.8*
Diabetes	1.1*	1.1*	1.1*	1.1*	1.1*	1.1*
Dengue fever (constrained)	1	1	1	1	1	1
TB	0.51*	0.65*	0.64*	0.69*	0.6*	0.67*
COVID-19	0.24*	0.097*	0.13*	0.26*	0.14*	0.25*
Variance	13	5.7	14	6.6	15	6.1
Likelihood ratio test (P value)	64.66 (< 0.001)					

*Z-test, P value < 0.001.

**Table 4:** Multigroup multilevel confirmatory factor analysis for residence, the Philippines, 2022.

Items	Unconstrained model				Constrained model	
	Urban		Rural			
	Within-households	Between-households	Within-households	Between-households	Within-households	Between-households
Cancer	0.65*	0.78*	0.68*	0.72*	0.67*	0.73*
Heart disease	0.95*	1.1*	0.77*	0.73*	0.83*	0.81*
Diabetes	1.1*	1.3*	1.1*	1.1*	1.1*	1.1*
Dengue fever (constrained)	1	1	1	1	1	1
TB	0.63*	0.64*	0.6*	0.67*	0.61*	0.67*
COVID-19	0.1*	0.46*	0.16*	0.23*	0.14*	0.26*
Variance	14	3.5	14	7.1	15	7
Likelihood ratio test (P value)	71.22 (< 0.001)					

*Z-test, P-value < 0.001.

## Discussion

The goal of the current study was to determine the structure of health knowledge as assessed by the PNDHS. Multilevel confirmatory factor analysis was conducted to assess the structure using two different models (two-level model, and variance model). It was found that all items contributed to health knowledge latent variable (Z-test, P < 0.001). However, the variance model showed that the variance within the households was greater than the variance between households (13 and 6.7, respectively).

The COVID-19 pandemic has become a severe health threat worldwide. A survey aimed to identify the knowledge about COVID-19 among the Filipino population revealed that 67.7% of the participants had low knowledge. However, the findings showed that females had greater knowledge than males (68.9% and 65.7%, respectively), and this difference was statistically significant (t-test, P < 0.001)^18^. In the current study, no measurement invariance was confirmed for gender. This may lead to that every group within the gender have different factor loadings on both latent variables. Notably, there was an obvious difference in the factor loading of knowledge of COVID-19: for males, the within-household loading was 0.24, and the between-household loading was 0.097; for females, the within-household loading was 0.13, and the between-household loading was 0.26. However, the constrained model shows that the within-household loading is 0.14 and that the between-household loading is 0.25.

Cardiovascular disease (CVD) has emerged as a new public health emergency in the Philippines^19^. CVD risk was greater in urban areas than in rural areas^20^. Regarding the current study, no measurement invariance was confirmed for residence, and a variation in the factor loading of knowledge of heart disease was observed: for urban areas, the within-household loading was 0.95 and the between-household loading was 1.1; for rural areas, the within-household loading was 0.77 and the between-household loading was 0.73. On the other hand, the constrained model shows that the within-household loading is 0.83 and the between-household loading is 0.81.

A scoping review was conducted to synthesize the status of educational interventions regarding diabetes. Overall, six studies showed that educational interventions significantly impacted self-efficacy, anthropometric measures, haemoglobin A1c levels, utilization of care and routine programmes, and attitudes towards health^21^. Similarly, in the current research, knowledge about diabetes had the maximum influence on the two latent variables.

Knowledge about cancer is crucial for reducing cancer morbidity and mortality in the Philippines, and poor knowledge of cancer screening is associated with low screening uptake^22^. Regarding current models, the loading of knowledge of cancer and TB is lower than that of knowledge of heart disease and diabetes.

In multilevel data, observations are correlated rather than independent, so the fundamental independence assumption that is required in statistical techniques, including “factor analysis”, is violated. As a result, the power of the statistical significance tests will be affected, and biases in parameter estimates and standard errors will be observed^23^. Multilevel confirmatory factor analysis is necessary when dealing with multilevel data. However, some studies have applied single-level modelling approaches for analysing such data^24^, while others have used multilevel confirmatory factor analysis^25,26^.

The GSEM is a flexible tool when we work with multilevel data, as multilevel latent variables (random effects assumed to have a Gaussian distribution whose realizations are at the level of a grouping variable) can be represented by a double circle in the SEM component^15^, but this option is not feasible in other software such as AMOS^27^.

The limitation of the current study is that the GSEM cannot provide goodness-of-fit statistics; therefore, comparative fit index (CFI), root mean squared error of approximation (RMSEA), or standardized root mean squared residuals (SRMR) cannot calculated. Therefore, the significance of loading was an indication of the importance of each item in the latent variable. However, the strength was that our study was based on national survey data (PNDHS, 2022).

## Data Availability

The data can be downloaded freely through a data request application from the Demographic Health Survey (DHS) website, URL: https://dhsprogram.com/data/available-datasets.cfm.
